# Perioperative fluid management: moving toward more answers than questions—a commentary on the RELIEF study

**DOI:** 10.1186/s13741-019-0113-3

**Published:** 2019-07-15

**Authors:** Timothy E. Miller, Rupert M. Pearse

**Affiliations:** 1 Duke University School of Medicine, Durham, NC USA; 2 Queen Mary University of London, London, UK

**Keywords:** Fluid management, Fluid restriction, Acute kidney injury

## Abstract

Perioperative fluid and hemodynamic management have been much-debated topics over the last few years. Recently, a number of large trials have been published to help inform this debate. The Restrictive versus Liberal Fluid Therapy for Major Abdominal Surgery (RELIEF) study is the largest trial to date of perioperative fluid management. The 3000-patient trial comparing 2 different fluid regimes showed that a restrictive fluid regimen during and up to 24 h after surgery was associated with an increase in acute kidney injury (AKI). This result is at odds with a recent trend to a more restrictive fluid approach during major surgery and suggests that practice may have become too restrictive. A moderately liberal (aiming for 1–2 l positive) or goal-directed approach is therefore recommended.

## Main text

Optimal perioperative fluid and hemodynamic management remain topics of significant debate in perioperative medicine. One reason for this is that perioperative care has changed significantly over the last 10 years as approaches such as laparoscopic surgery and enhanced recovery after surgery (ERAS) pathways have become routine. Much of the older clinical research in this field has lost relevance and new questions need to be answered. Another reason is that although we have had many trials of fluid and hemodynamic management over the years, most have been small single-center studies rather than large pragmatic multicenter trials. Fortunately, and thanks to the hard work of leading investigators worldwide, this has changed significantly over the last few years with the publication of a number of large multicenter studies on these topics (Calvo-Vecino et al. 2018; Futier et al. 2017; Pearse et al. 2014).

The Restrictive versus Liberal Fluid Therapy for Major Abdominal Surgery (RELIEF) study published in the New England Journal of Medicine in May 2018 is the largest trial published to date on perioperative fluid management (Myles et al. 2018). RELIEF was a 3000 patient international, multicenter, pragmatic randomized controlled trial comparing two different fluid regimes over 24 h in major abdominal surgery. The study included patients undergoing major abdominal surgery, both open and laparoscopic, with an expected duration of > 2 h, and an expected hospital stay greater than 3 days. The restrictive fluid intervention was designed to achieve a net zero fluid balance, with a 5 ml/kg bolus at induction of anesthesia followed by an intraoperative crystalloid infusion at a rate of 5 ml/kg/h, continued after surgery at 0.8 ml/kg/h for 24 h. In contrast, the liberal group received a 10-ml/kg bolus at induction of anesthesia followed by an intraoperative crystalloid infusion at a rate of 8 ml/kg/h, continued postoperatively at 1.5 ml/kg/h for 24 h. There was a clear separation in fluid administered between the treatment arms, suggesting successful delivery and implementation of the trial interventions. The restrictive regimen led to a median of 1.7 L of fluid administered intraoperatively with a weight gain of 0.3 kg, compared with 3 L intraoperative fluid and a weight gain of 3 L with the liberal regimen. The primary outcome of disability-free survival at 1 year was no different between the two groups. However, patients in the restrictive group had a significantly higher incidence of acute kidney injury (8.6% vs. 5%), renal replacement therapy (0.9% vs. 0.3%), and surgical site infection (16.5% vs. 13.6%). The findings of the RELIEF trial tell us that a restrictive fluid regime aimed at zero balance was associated with harm, especially acute kidney injury.

At first glance, this appears at odds with previous work suggesting the benefits of a restrictive fluid regime; however, much of this confusion relates to terminology, with imprecise meanings of the terms “restrictive” and “liberal.” The most widely known study advocating a restrictive fluid management approach by Brandstrup et al. was conducted in 2003 (Brandstrup et al. 2003). The study showed the clear superiority of the “restrictive” group; however, the harm signal occurred in the liberal group that received just over 6 L of fluid on the day of surgery with a postoperative weight gain of approximately 4 kg. The restrictive group in the Brandstrup study actually more closely resembles the liberal group in RELIEF with a weight gain of around 1 kg. This difference may reflect differing cultures of perioperative care in different countries or it may simply be that fluid management has evolved in the 15 years since Brandstrup’s work. With the advent of ERAS pathways, fluid management for major abdominal surgery has become more and more restrictive (Miller et al. 2015). The new learning from RELIEF appears to be that being too restrictive can also cause harm, particularly to the kidneys.

The RELIEF trial was well designed and well conducted with a low risk of bias. However, as is appropriate after an important trial is published, there will be a period of debate as the trial results are interpreted. One concern is that fewer than half of included patients were treated within a fully established enhanced recovery pathway although this may simply reflect international variations in usual care. Another concern is that fluid management should be individualized with goal-directed therapy (GDT) rather than using a standardized fluid algorithm. RELIEF was a pragmatic trial and clinicians were allowed to use ERAS y and goal-directed therapy pathways according to local practices. The fact that many centers did not use ERAS pathways in many ways reflects current practice with real-world implementation of ERAS being much lower in practice than in the literature, with many centers also overrating their ERAS compliance. Subgroup analyses show that the use of GDT and ERAS did not affect the primary results, suggesting that the findings are generalizable.

## Conclusions

We believe that the findings of the RELIEF trial will change practice for patients undergoing major surgery. There are times during any major surgical procedure when there is uncertainty regarding the risks and benefits of further fluid administration. This is true both for low-risk patients without advanced hemodynamic monitors and higher risk patients undergoing goal-directed therapy. In recent years, clinicians may have become less likely to give a fluid bolus when they are uncertain of the best course of action. RELIEF may have redressed our equipoise. The risks and benefits of intravenous fluid seem likely to remain a topic of discussion for some time to come.

## Abbreviations

ERAS: Enhanced recovery after surgery
GDT: Goal-directed therapy
RELIEF: Restrictive versus Liberal Fluid Therapy for Major Abdominal Surgery
